# Wastewater-based surveillance in Italy leading to the first detection of *mcr-10*-positive *Klebsiella quasipneumoniae*

**DOI:** 10.1186/s13756-022-01194-9

**Published:** 2022-12-09

**Authors:** Nicoletta Formenti, Flavia Guarneri, Cristina Bertasio, Giovanni Parisio, Claudia Romeo, Federico Scali, Laura Birbes, Maria Beatrice Boniotti, Giuseppe Diegoli, Loredana Candela, Gianluca Antonio Romeo, Paolo Pasquali, Giovanni Loris Alborali

**Affiliations:** 1 Istituto Zooprofilattico Sperimentale Della Lombardia e dell’Emilia Romagna “Bruno Ubertini”, Brescia, Italy; 2 Regione Emilia Romagna - Settore Prevenzione Collettiva e Sanità Pubblica, Bologna, Italy; 3 Ministero della Salute - Direzione Generale della Sanità Animale e dei Farmaci Veterinari, Rome, Italy; 4 Istituto Zooprofilattico Sperimentale dell’Abruzzo e del Molise “G. Caporale”, Teramo, Italy; 5 Istituto Superiore di Sanità - Dipartimento di Sicurezza Alimentare, Nutrizione e Sanità Pubblica Veterinaria, Rome, Italy

**Keywords:** Antimicrobial resistance, Colistin, Wastewater, Plasmid gene, AMR surveillance, *Klebsiella quasipneumoniae similpneumoniae*

## Abstract

Wastewater-based surveillance enabled the first detection of the mobile colistin resistance gene *mcr-10* in Italy. This plasmid-borne resistance gene was found in strains of *Klebsiella quasipneumoniae* isolated from samples of human raw sewage collected over several months. Although the isolates were phenotypically susceptible to colistin, the emergence of *mcr-10* is concerning due to the highly variable expression of the gene and the potential for horizontal transfer to other species. In addition, the strains also carried an extended-spectrum β-lactamase gene and were phenotypically resistant to several beta-lactams. This study highlights the value of wastewater-based surveillance as an effective tool to monitor the emergence of antimicrobial resistance in strains circulating in the community and the environment.

**Supplementary Information:**

The online version contains supplementary material available at 10.1186/s13756-022-01194-9.

## Background

In light of the global issue of antimicrobial resistance (AMR), implementing surveillance for the early detection of emerging resistances is paramount [1]. Although several new molecules have recently become available [2], colistin is still considered an important last-resort antimicrobial for the treatment of multidrug-resistant bacterial infections in humans, particularly in limited-resource settings. The recent emergence and global spread of plasmid-borne mobile colistin resistance (*mcr*) genes are therefore concerning, even because of their potential to spread rapidly among different bacterial species by horizontal transfer [1]. Wastewater-based surveillance (WBS) has recently emerged as a low-cost, real-time, and non-invasive way to track infection outbreaks in the human population [3, 4]. WBS has been widely applied in SARS-CoV-2 surveillance, but it applies to a variety of other pathogens and can potentially play a prominent role in guiding a timely public health response [3, 4].

Here we describe the first detection in Italy of the *mcr-10* gene, carried by strains of *Klebsiella quasipneumoniae*. The bacteria were isolated from human wastewater samples, highlighting the value of WBS as an effective tool for tracking new resistances in strains circulating in the community and the environment.

## Materials and methods

### Wastewater sampling and bacterial isolation

From November 2021 to May 2022, a total of 62 human 24 h composite samples of raw sewage (1 L each) were collected weekly from two lines of a wastewater treatment plant located in Northern Italy (Ferrara province, 44°50′07.07″N 11°37′11.51″E). The sampling was part of a larger research project on antibiotic resistance surveillance, mainly focussed on the detection of extended-spectrum β-lactamase (ESBL) genes. Thirty-one samples were collected from the city sewage purification line (L1; average flow rate: 257 L/s), while the other 31 samples came from the purification line of the city industrial pole (L2; an average flow rate of 212 L/s). The two lines have 120,000 population equivalents each. A volume of 100 ml of each wastewater sample was filtered using a vacuum filtration unit (model 514–0329, VWR International) with a 0.45 µm cellulose acetate membrane filter (VWR International). Next, filters were placed in buffered peptone water for the pre-enrichment phase. After overnight incubation, a drop of broth was used to inoculate MacConkey agar supplemented with 1 mg/L cefotaxime. Identification of all the phenotype-positive colonies was performed by biochemical tests (EnteroPluri-Test, Liofilchem) and by subsequent MALDI-TOF mass spectrometry (MS) (Vitek MS Plus, Biomerieux) methodology. All the strains were subjected to Minimum Inhibitory Concentrations (MICs) technique (Sensititre Vizion Digital MIC, Thermo Fisher Scientific), using a commercial panel for colistin (Sensititre FRCOL plates, Trek diagnostics, Thermo Fisher Scientific) and a commercial plate specific for surveillance on ESBL-producing isolates (Sensitre EUVSEC2 plates, Trek diagnostics, Thermo Fisher Scientific). The strains were classified as susceptible or resistant based on epidemiological cut-off values (ECOFFs, www.eucast.org, accessed on 3 November 2022) recommended by the European Committee on Antimicrobial Susceptibility Testing (EUCAST). EUCAST does not provide ECOFFs for all antibiotics included in the Sensititre EUVSEC2 plates. In the absence of ECOFFs for *K. pneumoniae*, those for *E. coli*, reference species for Enterobacteriaceae, were applied [5].

### Detection and sequencing of *mcr* genes

A single bacterial colony from each phenotype-positive strain was selected for *mcr* genes detection by PCR. Colonies were resuspended in 250 µL of DNase-RNase-free water, and DNA was extracted by lysis boiling (98 °C for 10 min). Two multiplex end-point PCR were set up for mcr 1 to 5 and 6 to 10, respectively. Available protocols were used for *mcr* alleles from 1 to 9 [6, 7], while for *mcr-10* the primers were designed based on the first submitted sequence [8]. For primer sequences and PCR reaction protocols see Additional file 1: Table S1.

Bacterial genomic DNA was extracted from the *mcr-10* positive isolates using the Nucleospin Tissue kit (Macherey Nagel). Libraries were prepared using the Illumina DNA Prep (M) Tagmentation kit (Illumina) and sequenced on the Illumina MiniSeq platform (2 × 150 bp). Reads were trimmed with Trimmomatic v0.39 [9] and assembled de novo utilizing SPAdes v3.15.4. The quality of the reads and the assemblies were assessed using FastQC v0.11.9 (https://www.bioinformatics.babraham.ac.uk/projects/fastqc/) and Quast v5.2 [10], respectively. Species identification and multi-locus sequence typing (MLST) were performed using Pathogenwatch platform (https://pathogen.watch). Resistance genes and plasmid replicons were identified using Resfinder v4.1 and PlasmidFinder v2.1 tools, respectively (http://www.genomicepidemiology.org/services/). Plasmid contigs were predicted using plasmidSPAdes [11] and by depletion of reads that mapped to the chromosome of a reference genome (*Klebsiella quasipneumoniae*: GenBank Accession Number NZ_CP065838) with Bowtie2 algorithm [12], in Geneious Prime software v11.0.14.1 + 1, and by assembling the unmapped reads via SPAdes. The presence of the *mcr-10* gene in the plasmid contigs was investigated using BLASTn [13]. Open Reading Frames (ORFs) of contigs containing *mcr-10* were found with Geneious Prime software and annotation was predicted by BLAST using NCBI Protein Reference sequences database, with one maximum hit and a similarity cut-off of 94%. The sequences of the detected *mcr-10*.1 genes are available in GenBank under Accession Numbers OP219652, OP219653, and OP219654. Raw reads of the three strains were submitted to the SRA database under the BioProject ID PRJNA892272.

## Results

A total of 22 isolates were found in the 62 wastewater samples collected during the study period. The isolates included 14 strains of *Escherichia coli*, four *Escherichia fergusonii*, and four strains of *Klebsiella* spp.

Three strains of *Klebsiella* spp. isolated from three different wastewater samples (3/62; prevalence = 4.8%; approximately 150 colonies/positive plate) were found to carry the *mcr-10* gene by PCR. No other *mcr* genes were detected during the study period. The three strains (hereafter R55, R58, R73) were isolated from samples collected from the industrial line (L2) in March (last week, R55), April (first week, R58), and May (second week, R73) 2022. MS initially identified the strains as *K. pneumoniae*. Sequencing analyses confirmed that the isolates belonged to the *K. pneumoniae* complex and further classified them as *Klebsiella quasipneumoniae* (subsp. *similpneumoniae*). The MLST analysis showed the presence of a novel *mdh* allele, identical to allele 26 except for one silent C3T mutation, leading to the undefined sequence type ST*3b12 (see Additional file 1: Table S2, for the complete allele profile).

Based on the *in-silico* analysis, the *mcr* gene, identical among the tested strains, was identified as an *mcr-10*.1 gene with 99.94% nucleotide identity to the first reported *mcr-10*.1 (*Enterobacter roggenkampii* strain 090,065, GenBank Accession Number MN179494), with one synonymous mutation (G1338A). The same single-nucleotide mutation was detected after comparison with the two other deposited plasmid-borne *mcr-10* sequences isolated in *K. quasipneumoniae*, referring to a water sample collected in 2000 in the Netherlands (GenBank Accession Number CP084774) and to a human clinical isolate sampled in 2015 in China (China National GeneBank DataBase Accession Number CNP0001198, [14]).

**Fig. 1:**
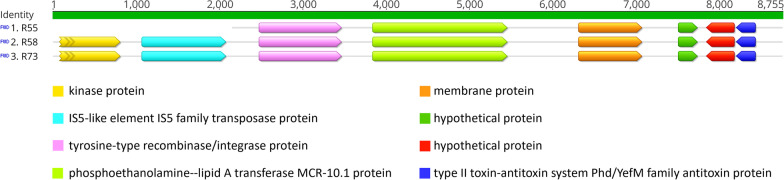
Genetic environment surrounding *mcr-10.1*—Alignment of the *mcr-10.1*-containing contigs of the three *Klebsiella quasipneumoniae* strains isolated from wastewater. Annotated regions are shown as oriented arrows according to the coding direction. Predicted functions are indicated with colour-coded keys

The *in-silico* analysis conducted on our strains, via plasmidSPAdes and by depletion of chromosome-associated reads, indicated that the *mcr-10* genes were located on plasmids. Sequencing indicated the presence of plasmid replicons IncFII(K) and IncR in all three strains, with R58 and R73 further carrying IncFIB(K). However, since the genes and the replicons were located on different contigs, we were unable to determine the specific replicon types associated with *mcr-10*. The nucleotide sequence located upstream of *mcr-10* showed the highest identity (94.85%) with several sequences (e.g. CP055065) characterized as tyrosine-type recombinase/integrase genes and isolated from *Enterobacter* spp. carrying *mcr-10* (Fig. 1). An insertion sequence (IS5 family) was located further upstream.

Despite the presence of *mcr-10*, the three strains were phenotypically susceptible to colistin by MIC (≤ 0.12 mg/L; Table 1). However, they also carried the ESBL gene *bla*_ACC-1_ and showed resistance to several 3rd and 4th generation cephalosporins, temocillin, and (only in strain R55) even mild resistance to ertapenem (Table 1).

**Table 1:** Minimum inhibitory concentrations (MICs) for the three *Klebsiella quasipneumoniae* strains R55 (March), R58 (April), and R73 (May) isolated from wastewater samples. EUCAST epidemiological cut-offs for *K. pneumoniae* (ECOFFs, www.eucast.org, accessed on 3 November 2022) relative to the tested antimicrobials are reported

Antimicrobial	MIC values (mg/L)			ECOFF (R > mg/L)
	R55	R58	R73	
Colistin (COL)	≤ 0.12	≤ 0.12	≤ 0.12	2
Cefepime (FEP)	**2**	0.12	**0.5**	0.125
Cefotaxime (FOT)	**8**	**2**	**16**	0.25
Cefotaxime/clavulanic acid (F/C)	**8**	**2**	**2**	0.25*
Cefoxitin (FOX)	4	4	4	8
Ceftazidime (TAZ)	**64**	**16**	**16**	1
Ceftazidime/clavulanic acid (T/C)	**32**	**16**	**32**	1
Ertapenem (ETP)	**0.06**	0.03	0.03	0.03*
Imipenem (IMI)	1	0.5	0.5	1
Meropenem (MERO)	0.06	0.06	≤ 0.03	0.125
Temocillin (TRM)	**16**	**16**	**32**	8

*in the absence of cut-offs for *K. pneumoniae*, values for *Escherichia coli* have been used

Resistances are highlighted in bold

## Discussion

The study describes the presence of three strains of *K. quasipneumoniae* (subsp. *similpneumoniae*) carrying *mcr-10* isolated from human wastewater samples. Although other colistin resistance genes had already been reported in Italy both in human and animal samples (e.g. 15, 16), to the best of our knowledge this is the first detection of *mcr-10* in the country. This specific colistin resistance gene has been so far isolated mostly in *Enterobacter* spp. [8, 17, 18], but was described also in *Klebsiella* spp. [14, 19] and in *E. coli* [20], showing, like other *mcr* genes, the potential for a wide host range. Because of the once heavy use of colistin in veterinary medicine, *mcr* genes are mostly found in bacterial isolates of animal origin [1] and more rarely in humans [14, 21], where colistin usage is instead limited. In a recent study, Liu et al. [14] reported a 0.7% prevalence of *mcr* genes after screening the genomes of almost 3000 clinical *Klebsiella* spp. isolates. Their analysis showed that *mcr*-carrying plasmids from human and nonhuman sources share many genetic elements, suggesting a wide circulation of these genes, as confirmed by our finding in wastewater.

*Klebsiella quasipneumoniae* is a potentially highly-virulent bacteria known to carry several AMR genes, including other *mcr* genes (e.g. 22), that was only recently described as a distinct species from *K. pneumoniae* and has been since isolated in both humans and animals worldwide [23]. In our case, within the 7-months study period, *K. quasipneumoniae* was isolated exclusively in those three consecutive months (March, April, and May) and all the isolated strains carried *mcr-10*. Moreover, all the isolates were found in samples from the industrial wastewater purification line (L2), suggesting that the carrier/carriers were workers or clients of the industrial pole who did not reside in the city. It must be noted, however, that using a medium selective for beta-lactam resistance could have led to an underestimation of the circulating *mcr-10*-positive strains, as only strains carrying both resistances would have been detected. The three isolated strains were indeed also phenotypically and genetically resistant to several beta-lactams, including in one case a slight resistance to a carbapenem, another last-resort antimicrobial. To the best of our knowledge, only two other plasmid-borne *mcr-10* sequences have been reported in *K. quasipneumoniae* so far, one in a strain isolated from a water sample collected in the early 2000s in the Netherlands (GenBank CP084774), and the other in a human clinical isolate from 2015 in China [14]. Our *mcr-10.1* sequences are identical to these sequences except for a single synonymous mutation, and neighbouring coding regions are also similar, although only at the family level. Most studies describe a recurrent genetic context located upstream from the gene and constituted by an insertion sequence (IS) and a gene coding for an integrase/recombinase, likely involved in *mcr-10* mobilization [8, 20, 21]. We found a consistent pattern, but in our case the IS and the integrase/recombinase did not match with those reported by most authors (i.e. IS26-family, IS903 and XerC).

Finally, despite carrying the *mcr* gene, the three *K. quasipneumoniae* isolates were all susceptible to colistin by MIC. Several studies reported a lack of phenotypical resistance to colistin in *mcr-10*-positive bacteria [8, 17, 19], but other authors found high levels of resistance [18, 20, 21], suggesting that *mcr-10* expression is largely dependent on the genetic context of the gene. For instance, Wang et al. [8] obtained a colistin-resistant *E. roggenkampi* strain when transferring *mcr-10* from a susceptible strain by cloning. Expression data by Guan et al. [18] and Xu et al. [21] further suggest that other, chromosomic genes might mediate the high level of colistin resistance observed in their *Enterobacter* spp. isolates. For this reason, the emergence of *mcr-10* in the area warrants attention: although our strains do not show phenotypical resistance to colistin, they might evolve resistance in the future, or the same gene might confer resistance when transmitted to other strains or bacterial species. Unfortunately, having no information about the specific plasmid harbouring our *mcr-10*, at present we cannot draw any conclusions regarding the transmissibility of the plasmid itself. However, there is increasing evidence that even nonconjugative plasmids can be transferred horizontally, either by involving helper plasmids or even through intermediate hosts such as *E. coli* [20, 24].

## Conclusions

We report for the first time the presence of the mobile colistin resistance gene *mcr-10* in Italy, detected in *K. quasipneumoniae* through wastewater-based surveillance. Although our isolates were not phenotypically resistant to colistin, the circulation of bacterial strains carrying plasmid-borne *mcr-10* is of concern due to the highly variable expression of the gene and the potential for horizontal transfer to other species. Besides, the identification of *mcr-10*-positive bacteria in wastewater highlights humans as potential carriers of *mcr* genes, despite their limited exposure to colistin. For these reasons, implementing ongoing surveillance for the early detection and monitoring of such genes is paramount. In this context, as highlighted by our findings, wastewater-based surveillance represents an invaluable tool to rapidly detect the presence of AMR genes and track their circulation in the population and the environment in a time- and cost-effective manner.

## Supplementary Information

**Additional file 1**.** Table S1**: Primers and PCR protocols for the detection of mobile colistin resistance (*mcr*) genes from 1 to 10 in bacteria isolated from wastewater samples. **Table** **S2**: Allele profile of R55, R58 and R73 strains of *Klebsiella quasipneumoniae* subsp. *quasipneumoniae* isolated from wastewater samples.

## Data Availability

Sequencing data of target genes have been deposited open access in public databases. Other raw data supporting the conclusions of this article will be made available by the authors upon request, without undue reservation.

## Abbreviations

AMR: Antimicrobial resistance
ECOFF: Epidemiological cut-off
ESBL: Extended-spectrum β-lactamase
EUCAST: European Committee on Antimicrobial Susceptibility Testing
MALDI-TOF: Matrix-assisted laser desorption ionization-time of flight
mcr: Mobile colistin resistance
MIC: Minimum inhibitory concentration
MLST: Multi-locus sequence typing
MS: Mass spectrometry
PCR: Polymerase chain reaction
ST: Sequence type
WBS: Wastewater-based surveillance
