# Supplementary material for: Wastewater-based surveillance in Italy leading to the first detection of mcr-10-positive Klebsiella quasipneumoniae
Source: Antimicrob Resist Infect Control. 2022 Dec 9;11:155. doi: 10.1186/s13756-022-01194-9 (PMC9734789; doi:10.1186/s13756-022-01194-9)
Supplement: Supplementary file 1 — Additional file 1. Table S1: Primers and PCR protocols for the detection of mobile colistin resistance (mcr) genes from 1 to 10 in bacteria isolated from wastewater samples. Table S2: Allele profile of R55, R58 and R73 strains of Klebsiella quasipneumoniae subsp. quasipneumoniae isolated from wastewater samples. [file 13756_2022_1194_MOESM1_ESM.pdf]

**Additional File 1** to:

# Wastewater-based surveillance in Italy leading to the first detection of *mcr-10*-positive *Klebsiella quasipneumoniae*

Nicoletta Formenti[1], Flavia Guarneri[1], Cristina Bertasio[1], Giovanni Parisio[1], Claudia Romeo[1,*], Federico Scali[1], Laura Birbes[1], Maria Beatrice Boniotti[1], Giuseppe Diegoli[2], Loredana Candela[3], Gianluca Antonio Romeo[3,4], Paolo Pasquali[5], Giovanni Loris Alborali[1]

*[1]Istituto Zooprofilattico Sperimentale della Lombardia e dell'Emilia Romagna "Bruno Ubertini", Brescia, Italy; [2]Regione Emilia Romagna - Settore Prevenzione Collettiva e Sanità Pubblica, Bologna, Italy; [3]Ministero della Salute - Direzione Generale della Sanità Animale e dei Farmaci Veterinari, Roma, Italy; [4]Istituto Zooprofilattico Sperimentale dell'Abruzzo e del Molise "G. Caporale", Teramo, Italy; [5]Istituto Superiore di Sanità - Dipartimento di Sicurezza Alimentare, Nutrizione e Sanità Pubblica Veterinaria, Roma, Italy; *corresponding author: Claudia Romeo (claudiarosa.romeo@izsler.it)*

**Supplementary Table 1.** Primers and multiplex PCR protocol for the detection of mobile colistin resistance (*mcr*) genes from 1 to 10 in bacteria isolated from wastewater samples.

| Target | Primer sequence (5'-3') | PCR mix | | Thermal profile | | |
|---|---|---|---|---|---|---|
| *mcr-1* | F: AGTCCGTTTGTTCTTGTGGC<br>R: AGATCCTTGGTCTCGGCTTG | DNA | 2 µL | 95°C | 5 min | x1 |
| *mcr-2* | F: CAAGTGTGTTGGTCGCAGTT<br>R: TCTAGCCCGACAAGCATACC | | | | | |
| *mcr-3* | F: AAATAAAAATTGTTCCGCTTATG<br>R: AATGGAGATCCCCGTTTTT | Dnase-free water | 6.75 µL | 95°C | 30 s | x30 |
| *mcr-4* | F: TCACTTTCATCACTGCGTTG<br>R: TTGGTCCATGACTACCAATG | | | | | |
| *mcr-5* | F: ATGCGGTTGTCTGCATTTATC<br>R: TCATTGTGGTTGTCCTTTTCTG | QIAGEN Multiplex PCR Plus Kit Master Mix (2x) | 12.5 µL | 60°C | 90 s | |
| *mcr-6* | F: AGCTATGTCAATCCCGTGAT<br>R: ATTGGCTAGGTTGTCAATC | | | | | |
| *mcr-7* | F: GCCCTTCTTTTCGTTGTT<br>R: GGTTGGTCTCTTTCTCGT | Primers premix (10 µM) | 1.25 µL | 72°C | 60 s | |
| *mcr-8* | F: TCAACAATTCTACAAAGCGTG<br>R: AATGCTGCGCGAATGAAG | | | | | |
| *mcr-9* | F: TTCCCTTTGTTCTGGTTG<br>R: GCAGGTAATAAGTCGGTC | Coral Dye | 2.5 µL | 72°C | 10 min | x1 |
| *mcr-10* | F: TGTTTGATCAGTCCATGATC<br>R: CGTGCCGTTTCGCCAATTA | | | | | |

**Supplementary Table 2.** Allele profile of R55, R58 and R73 strains of *Klebsiella quasipneumoniae* subsp. *quasipneumoniae* isolated from wastewater samples.

| **Gene** | ***gapA*** | ***infB*** | ***mdh*** | ***pgi*** | ***phoE*** | ***rpoB*** | ***tonB*** |
|---|---|---|---|---|---|---|---|
| **Allele** | 18 | 15 | novel | 16 | 11 | 13 | 51 |
